# Mass Detection and Segmentation in Digital Breast Tomosynthesis Using 3D-Mask Region-Based Convolutional Neural Network: A Comparative Analysis

**DOI:** 10.3389/fmolb.2020.599333

**Published:** 2020-11-11

**Authors:** Ming Fan, Huizhong Zheng, Shuo Zheng, Chao You, Yajia Gu, Xin Gao, Weijun Peng, Lihua Li

**Affiliations:** ^1^Institute of Biomedical Engineering and Instrumentation, Hangzhou Dianzi University, Hangzhou, China; ^2^Department of Radiology, Fudan University Shanghai Cancer Center, Shanghai, China; ^3^Computational Bioscience Research Center (CBRC), Computer, Electrical and Mathematical Sciences and Engineering Division (CEMSE), King Abdullah University of Science and Technology (KAUST), Thuwal, Saudi Arabia

**Keywords:** digital breast tomosynthesis, breast cancer, mass detection, deep learning, mass segmentation

## Abstract

Digital breast tomosynthesis (DBT) is an emerging breast cancer screening and diagnostic modality that uses quasi-three-dimensional breast images to provide detailed assessments of the dense tissue within the breast. In this study, a framework of a 3D-Mask region-based convolutional neural network (3D-Mask RCNN) computer-aided diagnosis (CAD) system was developed for mass detection and segmentation with a comparative analysis of performance on patient subgroups with different clinicopathological characteristics. To this end, 364 samples of DBT data were used and separated into a training dataset (*n* = 201) and a testing dataset (*n* = 163). The detection and segmentation results were evaluated on the testing set and on subgroups of patients with different characteristics, including different age ranges, lesion sizes, histological types, lesion shapes and breast densities. The results of our 3D-Mask RCNN framework were compared with those of the 2D-Mask RCNN and Faster RCNN methods. For lesion-based mass detection, the sensitivity of 3D-Mask RCNN-based CAD was 90% with 0.8 false positives (FPs) per lesion, whereas the sensitivity of the 2D-Mask RCNN- and Faster RCNN-based CAD was 90% at 1.3 and 2.37 FPs/lesion, respectively. For breast-based mass detection, the 3D-Mask RCNN generated a sensitivity of 90% at 0.83 FPs/breast, and this framework is better than the 2D-Mask RCNN and Faster RCNN, which generated a sensitivity of 90% with 1.24 and 2.38 FPs/breast, respectively. Additionally, the 3D-Mask RCNN achieved significantly (*p* < 0.05) better performance than the 2D methods on subgroups of samples with characteristics of ages ranged from 40 to 49 years, malignant tumors, spiculate and irregular masses and dense breast, respectively. Lesion segmentation using the 3D-Mask RCNN achieved an average precision (AP) of 0.934 and a false negative rate (FNR) of 0.053, which are better than those achieved by the 2D methods. The results suggest that the 3D-Mask RCNN CAD framework has advantages over 2D-based mass detection on both the whole data and subgroups with different characteristics.

## Introduction

Breast cancer is the most common malignancy in women. Full-field digital mammography (FFDM) is commonly used to screen for breast cancer (Nystrom et al., 2002). However, mammography has an inherent limitation when tissue overlaps, especially in dense breasts, which causes mammography to miss some suspicious cancerous lesions (Carney et al., 2003). Digital breast tomosynthesis (DBT) is an emerging breast cancer screening and diagnostic modality that takes quasi-three-dimensional imaging that can be used to provide a detailed assessment of the dense tissue within the breast. DBT has decreased the effect of overlapping tissue on screening, thereby improving lesion detection, characterization and diagnosis and making this approach superior to digital mammography (DM) (Michell et al., 2012; Haas et al., 2013). The integration of DBT into the diagnostic setting is associated with improved diagnostic performance of breast cancer due to the increased specificity (Bahl et al., 2019; Conant et al., 2019). The combination of DBT and mammography resulted in significant gains in the sensitivity and specificity of cancer detection compared with DM alone (Fontaine et al., 2019; Li et al., 2019; Skaane et al., 2019). Due to its improvements in patient diagnosis efficiency, DBT is becoming the standard of care in both screening and diagnostic breast images (Chong et al., 2019).

Early detection of masses on DBT can facilitate improved treatment and management in breast cancer. Additionally, segmentation of breast masses from the background tissue is important for accurate mass characterization and interpretation. However, the increment of the 3D information of breast tissue for DBT also increases the image reading workload by 2-fold (Tagliafico et al., 2017). Manual detection/segmentation of the breast region is therefore becoming impractical under a large number of samples/slices. Consequently, there is a need for computational methods to assist in the evaluation of DBT, both to address the workload issues and to maximize the performance of cancer detection and segmentation.

To this end, studies developed computer-aided diagnosis (CAD) system in DBT to facilitate mass detection and/or segmentation in a clinical setting. The conventional CAD studies have focused on 2D analysis of the slices of DBT using a variety of hand-crafted features (Reiser et al., 2006; Varela et al., 2006). Previous study used classical seed region growing algorithm to enhance the contour of a mass from a given region of interest (ROI) with the ability to adaptively adjust the threshold value (Berber et al., 2013). A Gaussian mixture models based on handcrafted intensity and texture measures were developed to segment breast masses in DBT (Pohlmann et al., 2017).

Compared to the conventional CAD using handcrafted features, deep learning-based CAD methods, which are based on end-to-end learning using a large amount of data, have an important role in DBT (Geras et al., 2019) due to their accuracy and efficiency. The deep CAD framework is reported to achieve much better performance than that achieved by using handcrafted features to detect masses in DBT (Yousefi et al., 2018). Moreover, a layered pathway evolution method was proposed to compress a deep convolutional neural network (DCNN) to classify masses in DBT (Samala et al., 2018). Previous studies developed a CAD system for mass detection and diagnosis using a DCNN with transfer learning from mammograms (Samala et al., 2016, 2019). A U-net based deep architecture was utilized to automatically segment breast masses on DBT data (Lai et al., 2020). To improve efficacy and accuracy in deep learning-based mass detection/segmentation, recent studies used CAD system based on one of the most successful object detection method, Faster RCNN [24] on mammograms (Ribli et al., 2018) and DBT (Fan et al., 2019). The existing studies were mainly performed using a DCNN based on 2D slices of DBT images for mass detection/segmentation. Nevertheless, volumetric, higher-dimensional information are more complicated so as to capture more sufficient, high-level features from 3D images. However, whether the 3D deep learning methods are superior to the traditional mass detection methods remains unknown.

There is also controversy regarding the efficiency of CAD methods for detecting masses in DBT from patients with different characteristics. For example, the DBT increases the cancer detection rate but is less effective for women with extremely dense breasts (Vourtsis and Berg, 2019). A recent study reported that DBT enabled the detection of more cancers in all density and age groups compared with DM, especially cancers classified as spiculated masses and architectural distortions (Osteras et al., 2019). DBT and DM screening increased the detection rate of histologically favorable tumors compared with that attained by DM screening (Hofvind et al., 2018). Therefore, it is of great interest to evaluate and compare the performances of deep learning-based mass detection and segmentation methods using DBT in patients with various characteristics, including different age ranges, breast densities, mass shapes and mass sizes.

Here, we proposed a framework for a 3D-MaskRCCN-based CAD system extended from our previous work of Faster RCNN on 2D slices of DBT (Fan et al., 2019), for the detection and segmentation of breast masses. To evaluate the effectiveness of 3D mask detection, we compared the results of the 3D-Mask RCNN, 2D-Mask RCNN, and Faster RCNN on images from patients with different characteristics. Our study was performed to enhance the efficiency and effectiveness mass detection/segmentation with DBT data and to facilitate an improved understanding of the 3D deep learning-based methods on different types of breast cancers.

## Materials and Methods

### Histological Analysis

Malignant and benign tumors were determined by biopsies using histological analysis. The breast density was determined according to the Breast Imaging Reporting and Data System (BI-RADS) ACR categories and/or quantification, which ranged from 1 to 4. Breasts with up to 25% mammary gland parenchyma were classified as ACR 1 (almost entirely fat), and those with 26–50% gland parenchyma (average density) were defined as ACR 2. The breasts with 51–75% gland parenchyma were classified as ACR 3 and those with more than 75% gland parenchyma (high density) were classified as ACR 4. The ACR type 3 and 4 breasts were categorized as dense breasts while ACR type 1 and 2 breasts were categorized as non-dense breasts.

### Dataset

The imaging and clinical data were collected from Fudan University Affiliated Cancer Center with Institutional Review Board (IRB) approval. Table 1 shows the characteristics of the samples used in this study. A total of 364 samples were collected (the mean age was 52.31 years, the age ranged from 18 to 88 years, and the median age was 51 years). Among samples, 75 were benign and 289 were determined to be malignant tumors by biopsy. The dense and non-dense breasts represented 75.8 and 24.2% of the total samples, respectively. There were 123 round/oval, 113 spiculate and 128 irregular masses. The data were randomly separated into the training dataset (*n* = 201) to train a deep learning-based CAD system and the testing dataset (*n* = 163) to test the effectiveness of the CAD. There were no significant differences in the ages, histologic types, mass types, and breast densities of the training and the testing datasets (*p* > 0.05).

**TABLE 1:** Patient clinicopathological characteristics.

Characteristics	All (*n* = 364)	Training dataset (*n* = 201)	Testing dataset (*n* = 163)	*p*
**Age (Years)**				0.807
<40	47(12.91%)	22(6.04%)	25(6.87%)	
40–49	116(31.87%)	65(17.86%)	51(14.01%)	
50–59	96(26.37%)	54(14.84%)	42(11.54%)	
60–69	76(20.88%)	43(11.81%)	33(9.07%)	
>70	29(7.97%)	17(4.67%)	12(3.30%)	
**Histological type**				0.98
Benign	75(20.60%)	42(11.54%)	33(9.07%)	
Malignant	289(79.40%)	159(43.68%)	130(35.71%)	
Mass type				0.73
Round/oval	123(33.79%)	69(18.96%)	54(14.84%)	
Spiculate mass	113(31.04%)	59(16.21%)	54(14.84%)	
Irregular	128(35.16%)	73(20.05%)	55(15.11%)	
Breast density				0.63
Non-dense^1^	276(75.82%)	51(14.01%)	37(10.16%)	
Dense^2^	88(24.18%)	150(41.21%)	126(34.62%)	
**Tumor maximum diameter (mm)**				0.38
10 ≤ d < 30	267	154	113	
30 ≤ d < 50	337	184	153	
50 ≤ d	112	56	56	

*^1^ACR 1 and 2. ^2^ACR 3 and 4. The differences in the patient characteristics of the groups in the training and testing datasets were compared using a χ^2^ test.*

Craniocaudal (CC) and mediolateral oblique (MLO) view DBT images were acquired by a Selenia Dimensions TM unit (Hologic, American) using a total tomographic angular range of 20° with a 1° increment of rotation and 20 projection views (PVs). The number of slices ranged from 20 to 124 (mean = 62.70 and median = 62), and the number of slices containing a lesion ranged from 6 to 111 (mean = 34.85 and median = 32). For each slice, the in-plane resolution was 106 × 106 μm. The entire DBT data set included 716 views from 364 breasts with 364 masses. The breast mass sizes ranged from 10.15 to 140.90 mm (mean = 36.45 mm and median = 33.40 mm).

### Image Preprocessing

Digital breast tomosynthesis images were reconstructed into a unified spacing slice (1.0 mm) using the simultaneous algebraic reconstruction technique (SART) (Zhang et al., 2006). To save computational memory and avoid the calculation of large-scale convolutions for the background pixels in the deep learning-based CAD system, the skin and the background were excluded from the breast region using a dynamic multiple thresholding-based breast boundary method (Wu et al., 2010; Kus and Karagoz, 2012).

### Mask RCNN-Based CAD System

#### 3D-Mask RCNN Architecture

**FIGURE 1:**
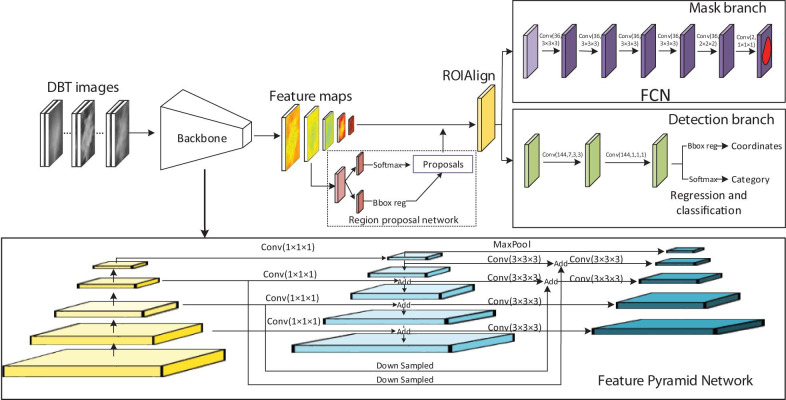
3D Mask RCNN architecture.

As an extension of the Faster RCNN (Ren et al., 2017) which performs the object detection of rectangular boxes as both a regression and classification problem task, Mask RCNN adds an additional branch that outputs the object mask (He et al., 2020). We developed a 3D-Mask RCNN-based breast mass detection and segmentation model, which is shown in Figure 1. Due to the substantial amount of memory required by the 3D convolution kernel in the network, we used small regions of the images (referred to as patches) with sizes of 256 × 256 × 64. The patches were used for training the 3D-Mask RCNN to obtain the mass detection model. Then, the model was applied to the patches in the testing set, which were subsequently recombined to reconstruct the entire DBT. The prediction probabilities of each patch are used to obtain the mass probability for the DBT, and the probable mass region was obtained by a bounding box.

The original Mask RCNN model was modified into the 3D version (Figure 1). In the network, the Residual Networks (ResNet)-Feature Pyramid Network (ResNet-FPN) backbone was used to extract different scales of the feature pyramid (He et al., 2016; Lin et al., 2017). FPN combines bottom-up features with top-down features in different scale. The ResNet has a structure of a depth of 50 layers in the 4th stage (ResNet-50-C4). This ResNet along with the FPN improve both the accuracy and speed of feature extraction. A region proposal network (RPN) was used to generate candidate bounding boxes from the input image. A quantization-free layer, i.e., RoIAlign was adopted to align the extracted feature maps with the inputs. This layer reduces misalignments between ROIs and the extracted features of RoIPool layer. The detection branch conducted mass detection for each proposed ROIs using a classifier network and bounding-box regression to obtain the probabilities and position information for the boxes. The mask branch obtained probabilities and position information from the feature maps and predicted a segmentation mask from each ROI using a fully connected network (FCN) in a pixel-to-pixel manner. The rectified linear unit (ReLU) were used as activation function in all the layers. The 3D-Mask RCNN were compared with 2D-Mask RCNN, Faster RCNN and Spatial Fuzzy C-Means (SFCM) (Zhang and Li, 2014) methods.

#### 3D-Mask RCNN Training

The hyperparameters of the networks including batch size, RPN train anchors, number of epochs, learning rate, and backbone are shown in Supplementary Table S1. To train the Mask RCNN, the network was initialized using the strategy proposed by He et al. (2015) which has good performance and was trained using the Adam optimizer (Kingma and Ba, 2014). For mass segmentation, the mask loss was defined only with the positive ROI. The initial learning rate was 0.001 and it was reduced by a factor of 0.5 after every 50 epochs. This learning rate was changed during training to achieve increased performance and faster training. Each mini-batch had 32 proposed ROIs. A mask branch for predicting an object mask was added to the RCNN. The same end-to-end training was performed to jointly train the RPN and the whole network.

In the training of the Faster RCNN, which was used in comparison with our framework, the weights were randomly initialized using a zero-mean Gaussian distribution. The initial learning rate determined by experiments was set as 0.001 for all the layers and was reduced by a factor of 0.5 after every 50 epochs. Each mini-batch had 2 images per GPU with 256 sampled ROIs. The loss function was divided into two parts: the first part was the classification loss, and the second part was the bounding box loss, with the Smooth L1 regularization defined in Ren et al. (2017). End-to-end training that jointly trains the RPN and Faster RCNN was performed to train the whole network.

### Performance Analysis

The detected target was compared with the true masses marked by an experienced radiologist. More specifically, an experienced radiologist manually annotated the 3D bounding boxes, and the true positive (TP) objects were represented by the ROIs extracted from the radiologist-marked locations. The background or non-mass regions were labeled as negative cases. For mass segmentation, the detection was determined to be a TP if its intersection over union (IOU) for the true masses was greater than 50%. The ratio of the positive to negative ROIs was 3 to 2.

We calculated a free-response receiver operating characteristic (FROC) curve defined as the plot of the sensitivity versus the average number of false positives per breast/lesion. The FROC curve was computed by varying the thresholds of the object prediction confidence (Bandos et al., 2010). The lesion-based FROC (the same lesion imaged in the CC and MLO views was regarded as different targets for detection) and the breast-based curve (the same lesion imaged in two views of a breast was considered to be one target and the detection of one or both was regarded as a TP) were both assessed. The average precision (AP) and false negative rate (FNR) were used to evaluate the effectiveness of the segmentation methods.

The comparisons of the performances of the two CAD systems were conducted by calculating the differences between the area under the FROC (Bornefalk and Hermansson, 2005; Bandos et al., 2009) using the Bootstrap test to resample the prediction score of the detection system under non-parametric assumptions. The statistical significance of the performance difference between our 3D-Mask RCNN and the other two 2D deep CAD systems was estimated based on the breast-based FROC curves.

Ten-fold cross-validation (CV) was used for the training dataset to tune the hyperparameters of the deep learning-based CAD system. In each CV cycle, the deep learning-based CAD systems were trained using nine subsets as the training set and one subset as the testing set. The hyperparameter with the best performance in the 10-fold CV was used to train the CAD system using all the samples in the training set. Then, the trained model was applied to the testing dataset to evaluate its effectiveness on mass detection/segmentation.

## Results

### Assessment and Comparison of Mass Detection Methods

#### Comparison of the Deep Mass Detection Methods on All Samples in Testing Set

**FIGURE 2:**
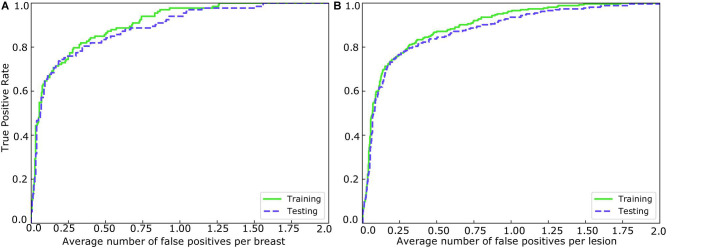
FROCs for the training and testing sets in **(A)** breast-based and **(B)** lesion-based mass detection.

**FIGURE 3:**
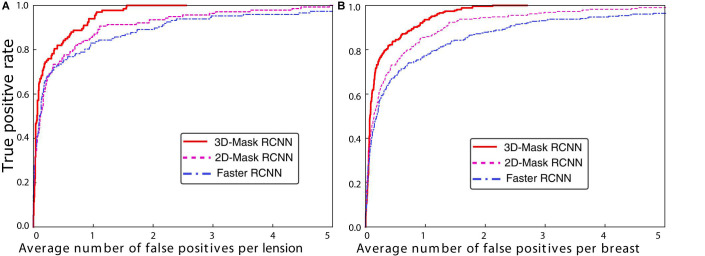
FROC curves for the 3D-Mask RCNN-, Faster RCNN- and 2D-Mask RCNN-based CAD systems in **(A)** lesion-based and **(B)** breast-based detection.

Mass detection was performed on all the samples in the testing set using the 3D-Mask RCNN, and the results were compared with those of the 2D-Mask RCNN and Faster RCNN (Figure 2). The results of the 3D-Mask RCNN CAD system achieved a sensitivity of 90% with 0.80 FPs/lesion and 0.83 FPs/breast, respectively. The mean numbers of FPs per breast at different sensitivities, which were determined based on the FROC curves, are shown in Table 2 and Figure 3. Our 3D-Mask RCNN-based CAD system clearly has better detection performance (in terms of fewer FPs) than that of the 2D-Mask RCNN or the Faster RCNN at all the sensitivities.

**TABLE 2:** Mean numbers of FPs per lesion and breast at different sensitivities from the FROC curves.

Sen (%)	3D DBT						2D DBT			
	3D-Mask RCNN		2D-Mask RCNN		Faster RCNN		2D-Mask RCNN		Faster RCNN	
	L	B	L	B	L	B	L	B	L	B
60	**0.10**	**0.08**	0.22	0.19	0.29	0.17	**0.35**	0.28	0.83	**0.17**
70	**0.16**	**0.22**	0.38	0.34	0.54	0.33	**0.72**	0.68	1.63	**0.33**
80	**0.33**	**0.34**	0.69	0.77	1.18	0.97	**1.25**	1.26	2.99	**0.97**
85	**0.57**	**0.57**	0.91	1.06	1.66	1.49	**1.95**	2.33	4.26	**1.49**
90	**0.80**	**0.83**	1.30	1.24	2.37	2.38	**2.77**	3.08	5.36	**2.38**
95	**1.10**	**1.02**	2.38	2.80	4.05	3.30	**4.25**	4.28	7.04	**3.30**

*Sen, sensitivity; L, lesion-based mass detection; B, breast-based mass detection. The lowest FP values for the mass detection methods are in bold.*

For these methods, the detection performances were compared, and statistical tests showed a significant difference in the areas under the breast-based FROC curves between the 3D-Mask RCNN and 2D-Mask RCNN methods (*p* = 0.005). Moreover, the 3D-Mask RCNN method achieved significantly better detection performance than that of the Faster RCNN-based system with a *p* value of 0.007. These results demonstrated that 3D-Mask RCNN-based CAD outperformed the 2D methods of the 2D-Mask RCNN and Faster RCNN (Supplementary Table S2).

In addition to the deep learning-based mass detection using 3D information of DBT data, the effectiveness of these methods is also examined on 2D slices of DBT. To this end, we evaluated the detection performances of the 2D-Mask RCNN and Faster RCNN using the imaging slice that shows the lesion with maximum diameter among that of all the DBT slices. The results showed that the Mask RCNN achieved better performance than the Faster RCNN in terms of fewer FPs/lesion, whereas an inverse result was observed for the breast-based evaluation, which showed fewer FPs/breast for the Faster RCNN compared with the Mask RCNN (Table 2). The two methods for both the lesion- and breast-based mass detection on 2D slices of DBT showed lower performance detection compared with that based on 3D volume of DBT (Table 2).

#### Comparison of the Deep Mass Detection Methods on Patients With Various Characteristics

**FIGURE 4:**
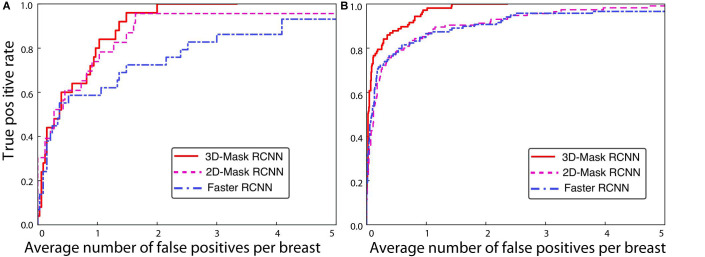
FROC curves for the 3D-Mask RCNN-, Faster RCNN- and 2D-Mask RCNN-based CAD systems for **(A)** benign and **(B)** malignant tumors.

**FIGURE 5:**
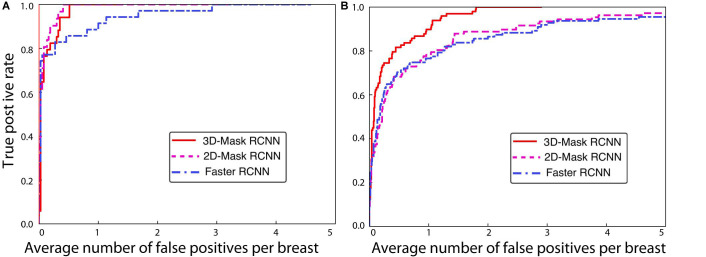
FROC curves for the 3D-Mask RCNN-, Faster RCNN- and 2D-Mask RCNN-based CAD systems for patients with **(A)** non-dense and **(B)** dense breasts.

**FIGURE 6:**
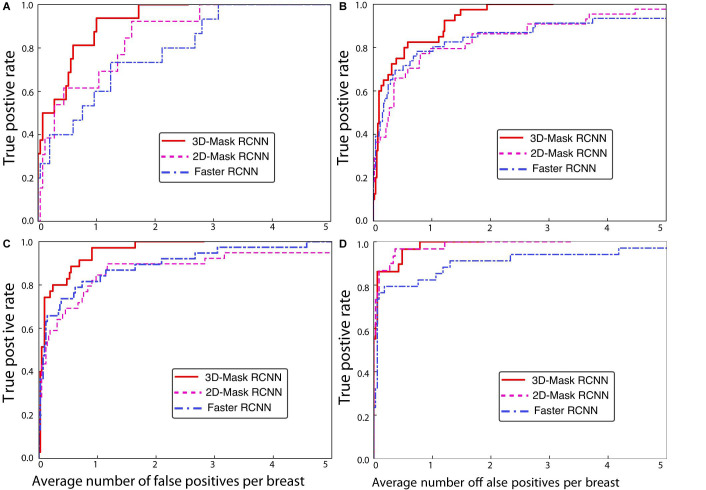
FROC curves for the 3D-Mask RCNN-, Faster RCNN- and 2D-Mask RCNN-based CAD systems for patients with ages **(A)** less than 40 years, **(B)** 40–49 years, **(C)** 50–59 years, and **(D)** 60–69 years.

A comprehensive comparison of the mass detection results of the 3D and 2D deep learning methods was performed on samples with different patient characteristics. The FPs per breast at a sensitivity of 90% for the CAD systems are shown in Table 3. The performances of the 3D-Mask RCNN, 2D-Mask RCNN and Faster RCNN on patients with different characteristics including benign/malignant tumors, breast densities and ages are shown in Figures 4–6, respectively.

**TABLE 3:** Comparison of the FPs of the 2D-Mask RCNN, 3D-Mask RCNN and the Faster RCNN-based CAD at a sensitivity of 90%.

Characteristic	2D-Mask RCNN^1^	Faster RCNN^1^	3D-Mask RCNN^1^	3D-Mask RCNN vs. 2D-Mask RCNN^2^	3D-Mask RCNN vs. Faster RCNN^2^
**Age**					
<40	1.6	2.8	**1.00**	0.291	0.1
40–49	2.62	2.76	**1.21**	**0.024**	**0.026**
50–59	2.83	2.09	**0.69**	0.100	**0.047**
60–69	0.27	1.3	0.48	**0.015**	0.062
>70	0	0.08	**0**	0.097	0.192
**Histological type**					
Benign	1.6	4.09	**1.36**	0.058	**0.029**
Malignant	1.28	1.76	**0.67**	**0.015**	**0.019**
**Mass type**					
Round/oval	0.78	1.2	**0.41**	0.061	**0.017**
Spiculate mass	0.9	1.7	**0.75**	**0.015**	**0.022**
Irregular	3	4.54	**1.20**	**0.023**	**0.016**
**Breast density**					
Non-dense	**0.18**	1	0.35	0.101	0.064
Dense	2.47	2.82	**1.03**	**0.005**	**0.010**
**Maximum diameter (mm)**					
10 ≤ d < 30	1.3	3.15	**0.77**	**0.007**	**0.030**
30 ≤ d < 50	1.26	2.24	**0.60**	**0.007**	**0.010**
d ≥ 50	1.98	2.71	**1.19**	**0.037**	**0.039**

*^1^False positive (FP) values at a sensitivity of 90%. ^2^P values are provided for comparisons of differences between the area under the free-response receiver operating characteristic (FROC). The lowest FP values and significant p values are in bold.*

The 3D-Mask RCNN achieved fewer FPs/breast for almost all the age ranges. For the patients with ages from 40 to 49 years, the 3D-Mask RCNN had significantly better performance than that of the 2D-Mask RCNN (*p* = 0.024) and the Faster RCNN (*p* = 0.026). Additionally, the mass detection performance in terms of the fewest FPs/breast was higher for malignant tumors than benign tumors for all the detection methods (Figure 4). The 3D-Mask RCNN also achieved significantly better performance in the detection of malignant tumors than the 2D-Mask RCNN and the Faster RCNN with *p* values of 0.015 and 0.019, respectively. All the methods achieved lower mass detection performance for irregular tumors than other mass types with the highest FPs/breast. Among the mass types, the 3D-Mask RCNN model achieved significantly better detection performance on spiculate or irregular masses than either the 2D-Mask RCNN or the Faster RCNN-based CAD system (*p* < 0.05).

Furthermore, it was observed that the detection performance is lower in samples with dense breasts than those with non-dense breasts in terms of low FPs for all the detection methods (Figure 5). The 3D-Mask RCNN achieved significantly better detection performance than the 2D-Mask RCNN- and the Faster RCNN-based (*p* = 0.005 and 0.010, respectively) CAD in patients with dense breasts. Furthermore, deep learning-based mass detection performs better for larger masses than smaller masses (Figure 6). Again, our 3D-Mask RCNN method has better mass detection performance than the other methods for all the diameter sizes (*p* < 0.05).

#### Case Study of the Mass Detection of the Deep Learning-Based Mass Detection Methods

**FIGURE 7:**
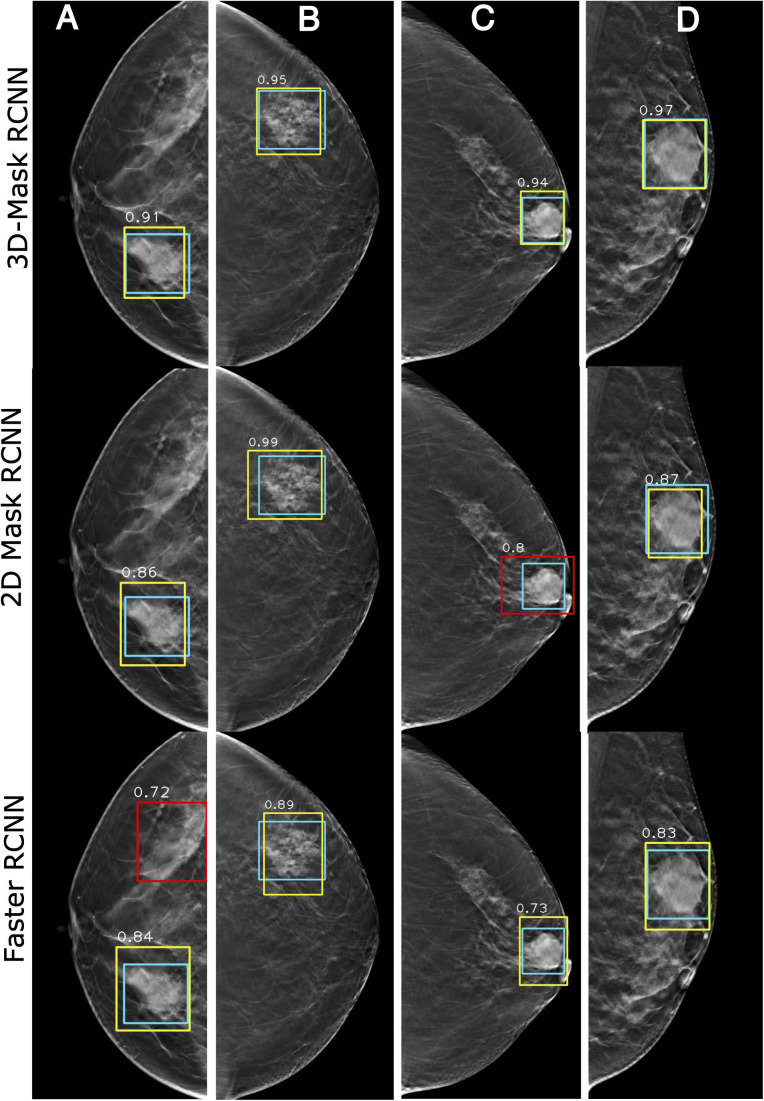
Examples of mass detection results using the 3D-Mask RCNN, 2D-Mask RCNN and Faster RCNN on patients with different densities. Four patients are included (from left to right) with the following characteristics: **(A)** aged 55 years, low density breast, malignant tumor, spiculate mass, and maximum tumor diameter of 43.24 mm; **(B)** aged 54 years, low density breast, malignant tumor, irregular mass, and maximum tumor diameter of 28.36 mm; **(C)** aged 69 years, low density breast, malignant tumor, oval mass, and maximum tumor diameter of 28.36 mm; and **(D)** aged 45 years, dense breast, malignant tumor, oval mass, and maximum diameter of 41.42 mm (green: ground truth box, yellow and red: detection box).

**FIGURE 8:**
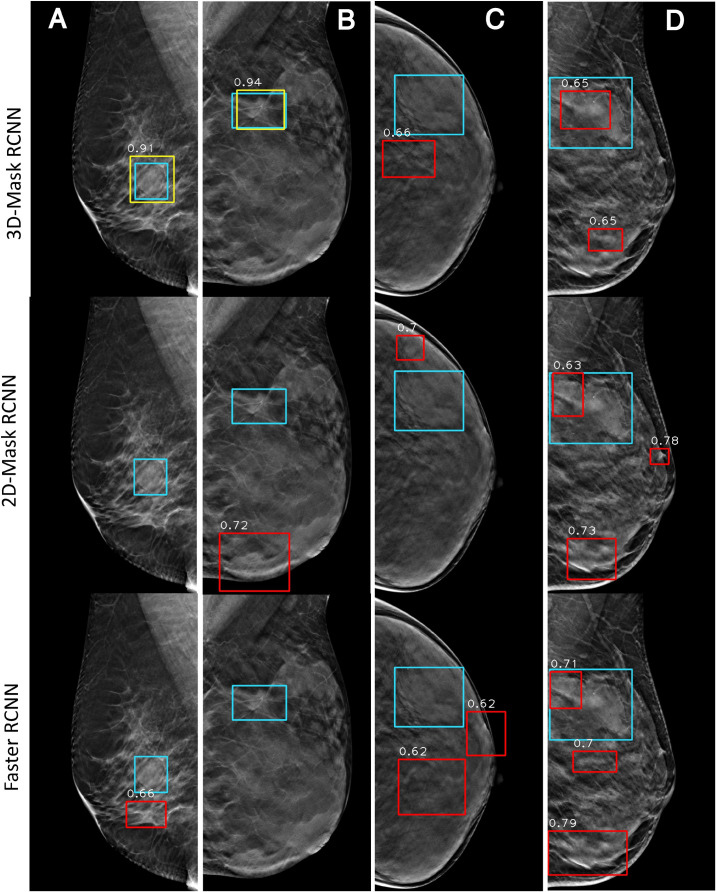
Examples of mass detection results for the 3D-Mask RCNN, 2D-Mask RCNN and Faster RCNN in patients with dense breast. Four patients are included (from left to right) with the following characteristics: **(A)** aged 60 years, dense breast, benign tumor, oval mass, and maximum tumor diameter of 24.62 mm; **(B)** aged 30 years, dense breast, malignant tumor, spiculate mass, and maximum tumor diameter of 38.35 mm; **(C)** aged 43 years, dense breast, malignant tumor, irregular mass, and maximum tumor diameter of 46 mm; and **(D)** aged 51 years, dense breast, malignant tumor, irregular mass, and maximum tumor diameter of 59.82 mm (green: ground truth box, yellow and red: detection box).

**FIGURE 9:**
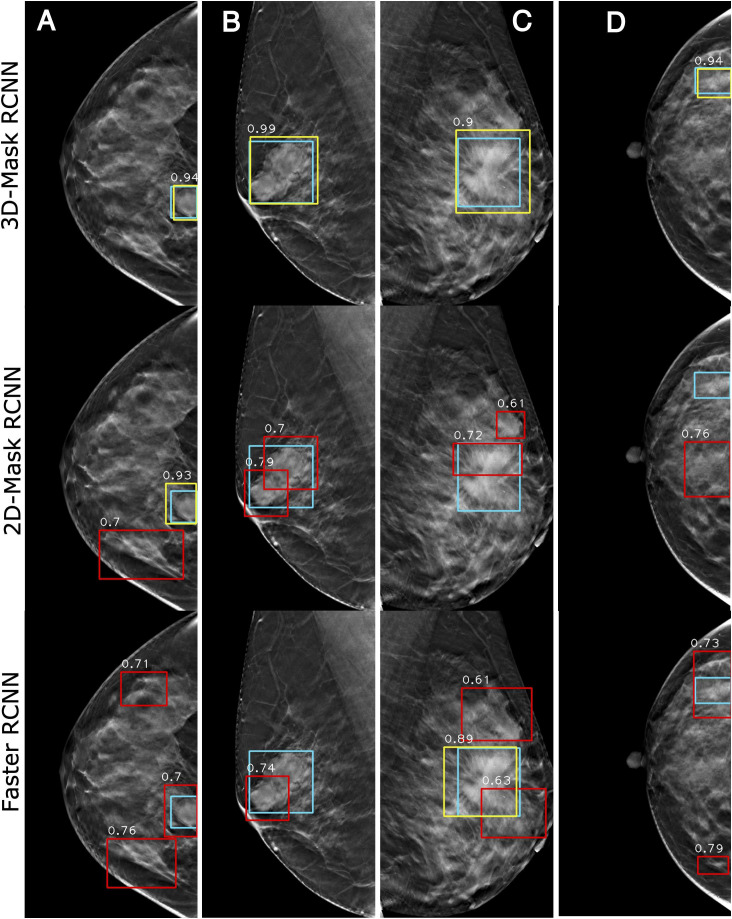
Examples of mass detection results for the 3D-Mask RCNN, 2D-Mask RCNN and Faster RCNN in patients with dense breast. Four patients are included (from left to right) with the following characteristics: **(A)** aged 33 years, dense breast, benign tumor, oval mass, and maximum tumor diameter of 24.24 mm. **(B)** aged 52 years, dense breast, benign tumor, spiculate lesion, and maximum tumor diameter of 50.35 mm; **(C)** aged 48 years, dense breast, malignant tumor, spiculate lesion, and maximum tumor diameter of 62.72 mm; and **(D)** aged 40 years, dense breast, benign tumor, irregular mass, and maximum tumor diameter of 26.58 mm (green: ground truth box, yellow and red: detection box).

Figure 7 shows the examples of the mass detection for the 3D-Mask RCNN, 2D-Mask RCNN and Faster RCNN methods. From this figure, tumors with low densities are easier for the CAD system to detect (Figures 7A–C). The three methods also showed high prediction scores on dense breast with characteristic of malignant, oval and small tumor (Figure 7D). However, the Faster RCNN method showed a false positive detection result (Figure 7C) while the 2D Mask RCNN showed lower detection score than the other methods (Figure 7A). Figure 8 illustrates the mass detection results in patients with dense breast, but with different age ranges, mass shapes and histological types. The results showed that 3D deep mass detection achieved better performance than those of the other two 2D methods, which failed to detect masses in patients with dense breasts and spiculate tumors. However, all three methods failed to detect the masses of patients with large lesion sizes and dense breasts (Figure 8C). Additionally, the results showed low detection scores for all the three methods on patients with large lesion sizes, dense breasts and irregular shapes (Figure 8D). Figure 9 illustrates the detection results for patients with dense breasts. Our 3D-Mask RCNN CAD system outperformed the other 2D methods on the four cases. The 2D-Mask RCNN and Faster RCNN achieved lower detection performance than the 3D-Mask RCNN in the detection of large tumors (Figures 9B,C). Compared with the 3D method, it is more difficult for the 2D or Faster RCNN to discriminate lesions and background regions in patients with dense breasts with smaller mass size (Figures 9 A,D).

### Assessment and Comparison of Mass Segmentation Methods on DBT

Lesion segmentation was performed using the 3D-Mask RCNN-, 2D-Mask RCNN- and SFCM-based clustering methods. Table 4 illustrates the tumor segmentation results for all the samples in the testing set and, in the subgroups according to the age range, histological type, mass type, breast density and lesion size. From the table, the 3D segmentation method clearly achieved superior performance compared with the 2D-Mask RCNN- and SFCM-based methods with higher APs and lower FNRs.

**TABLE 4:** Tumor segmentation results of the 3D-Mask RCNN, 2D-Mask RCNN and SFCM-based CAD method.

Characteristics	3D-Mask RCNN		2D-Mask RCNN		SFCM	
	AP	FNR	AP	FNR	AP	FNR
All	**0.934**	**0.053**	0.730	0.260	0.674	0.317
**Age**						
<40	**0.934**	**0.055**	0.734	0.255	0.684	0.307
40–49	**0.934**	**0.052**	0.731	0.259	0.671	0.320
50–59	**0.929**	**0.057**	0.756	0.233	0.676	0.315
60–69	**0.921**	**0.065**	0.743	0.246	0.689	0.303
>70	**0.950**	**0.036**	0.717	0.272	0.623	0.367
**Histological type**						
Benign	**0.937**	**0.049**	0.723	0.267	0.654	0.338
Malignant	**0.932**	**0.055**	0.735	0.254	0.658	0.332
Mass type						
Round/oval	**0.933**	**0.054**	0.722	0.267	0.670	0.321
Spiculate mass	**0.939**	**0.049**	0.717	0.271	0.687	0.303
Irregular	**0.931**	**0.055**	0.727	0.263	0.686	0.306
**Breast density**						
Low^1^	**0.931**	**0.055**	0.743	0.246	0.668	0.323
High^2^	**0.933**	**0.054**	0.727	0.262	0.669	0.322
**Maximum diameter (mm)**						
10 ≤ d < 30	**0.931**	**0.056**	0.734	0.255	0.668	0.323
30 ≤ d < 50	**0.930**	**0.056**	0.730	0.259	0.674	0.317
d ≥ 50	**0.935**	**0.052**	0.726	0.263	0.674	0.317

*AP, Average Precision; FNR, False Negative Rate. The highest AP or lowest FNR values for the mass detection methods are in bold.*

### Running Time Evaluation and Comparison

The training and testing of the deep CAD model were performed on a Linux workstation with 16 CPU cores (2.1 GHz) and 6 NVIDIA 1080Ti GPUs with 11 GB of memory. The execution time for the 3D-Mask RCNN, the 2D-Mask RCNN and the Faster RCNN were approximately 350, 260, and 245 h, respectively. The detailed description of the running times was illustrated in Table 5.

**TABLE 5:** Running time comparison of the deep mass detection networks.

Name	Size (MB)	Parameters	Time per image (ms)
2D-Mask RCNN	244	4.93e + 07	195
3D-Mask RCNN	320	5.27e + 07	100
Faster RCNN	533	1.41e + 08	210

## Discussion

The Mask RCNN framework was developed to detect masses in breasts. It has been shown that the 3D-Mask RCNN is superior to the other two deep learning-based methods, namely, the 2D-Mask RCNN and the Faster RCNN. Moreover, we have assessed the detection performance in various subgroups of patients with different age ranges, breast densities, histological types and tumor shapes. The results suggested that the 3D-Mask RCNN achieved significantly better performances than the 2D deep CNN models on specific groups according to clinicopathological features.

A previous study used the Faster RCNN model with VGG16 on the INBreast dataset to detect malignant masses and calcifications (Ribli et al., 2018). A deep CNN with multiple instance learning (Yousefi et al., 2018) achieved better performances than the handcrafted features-based CAD systems on 2D slices of DBT images. Samala et al. (2016) presented a DCNN-based approach for mass detection using DBT images. A recent study used the deep learning (Samala et al., 2019) method with transfer learning to discriminate between the malignant and benign masses in DBT images. However, these studies were performed based on the 2D analysis of a deep neural network. In this study, we showed that the 3D deep learning method is superior to the 2D methods in both mass detection and segmentation. It is interesting to note that the Mask RCNN has better lesion-based detection performance while the Faster RCNN achieved better breast-based mass detection in DBT images.

**FIGURE 10:**
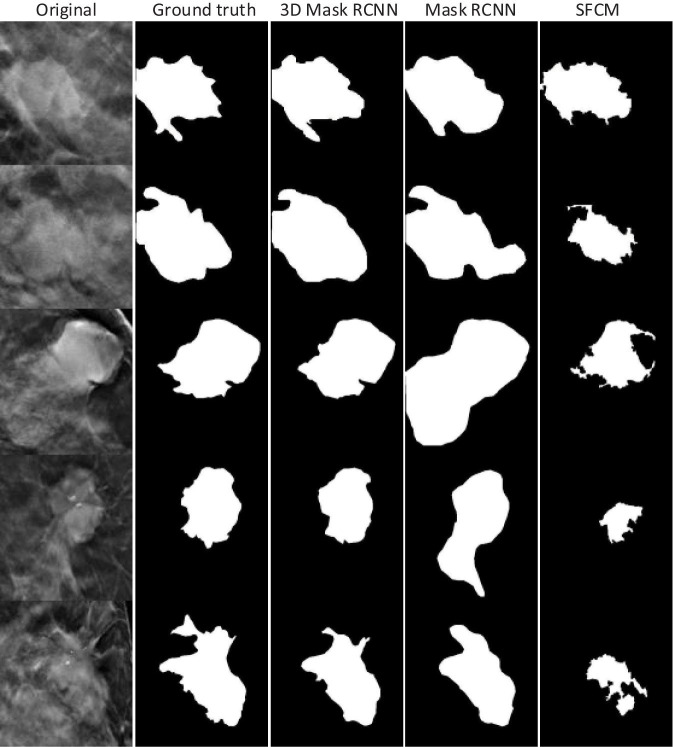
Tumor segmentation results for the 3D-Mask RCNN, 2D-Mask RCNN, and SFCM. Five patients are included (from top to bottom).

The systematic analyses of the CAD systems showed that the mass detection performances are correlated with patient characteristics, such as age, histological type, mass type, breast density, and mass size. The breast masses of patients who are 40–59 years old are more difficult to detect. Moreover, CAD detection was less accurate with more FPs for the samples with benign tumors, irregular shapes, and dense breasts. Smaller (10 ≤ d < 30) and larger (d ≥ 50) tumors were difficult for the deep learning-based detection methods to detect (Table 3). We observed that the 3D-Mask RCNN has significantly better (*p* < 0.05) mass detection performance than the other 2D methods, especially for specific groups that were more difficult to detect (i.e., those aged 40–59, benign tumors, irregular tumors and dense breasts). This may be explained by the fact that DBT reduces the tissue overlap and increases the lesion conspicuity, particularly in dense breasts, which makes it rational that 3D methods have better detection performance than those of the 2D methods. Moreover, 3D-Mask RCNN method take advantage of volumetric information of DBT, which is better than 2D methods in discriminating the masses of irregular shapes from normal tissues (e.g., fibroglandular) with fine textures/structures, especially in dense breasts. Since age is inversely associated with breast density (Checka et al., 2012), it is reasonable that detection performance is higher in older patients (aged 60 years and older) than that in the others. The 3D-Mask RCNN also achieved better segmentation performance than those of the 2D methods in the entire testing set and the subgroups (Table 4). The case study also illustrates that the 3D-Mask RCNN had fewer false positives than the other methods (Figure 10).

The limitations of this study should be addressed. First, the sample size in this study is relatively small, especially when the subgroup analysis was conducted on patients with different clinicopathological characteristics. A data cohort with a larger size should be used in the future to refine the results of our study. Second, we do not perform transfer learning in this study. A future study with transfer learning from mammograms might enhance the accuracy of the mass detection/segmentation in patients. Third, we used image patches for detection to save computer memory, and thus, future studies that focus on the entire image should be conducted.

## Conclusion

In summary, we proposed a 3D-Mask RCNN-based mass detection and segmentation framework for detecting and segmenting tumor masses. A comparison of the 3D- and 2D-based methods under different subgroups based on age ranges, lesion sizes, lesion shapes, and breast densities was conducted. We illustrated that the 3D-Mask RCNN has better performance than the 2D methods, especially for subgroups with specific clinicopathologic characteristics that show higher FPs, and the improvement is significant.

## Data Availability Statement

The original contributions presented in the study are included in the article/Supplementary Material, further inquiries can be directed to the corresponding authors.

## Ethics Statement

The studies involving human participants were reviewed and approved by Shanghai cancer hospital, Fudan University. Written informed consent for participation was not required for this study in accordance with the national legislation and the institutional requirements.

## Author Contributions

MF, WP, and LL designed the study. HZ, SZ, and CY performed the image processing, deep learning methods, and statistical analyses. MF and LL wrote the manuscript. YG and XG analyzed the data. All authors reviewed the manuscript.

## Conflict of Interest

The authors declare that the research was conducted in the absence of any commercial or financial relationships that could be construed as a potential conflict of interest.

## References

[B1] Bahl M. Mercaldo S. Vijapura C. A. McCarthy A. M. Lehman C. D. (2019). Comparison of performance metrics with digital 2D versus tomosynthesis mammography in the diagnostic setting. *Eur. Radiol.* 29 477–484. 10.1007/s00330-018-5596-7 29967957

[B2] Bandos A. Rockette H. T. Gur D. (2010). Area under the free-response ROC curve (FROC) and a related summary index. *Biometrics* 65 247–256. 10.2307/25502264 PMC2776072 18479482

[B3] Bandos A. I. Rockette H. E. Song T. Gur D. (2009). Area under the free-response ROC curve (FROC) and a related summary index. *Biometrics* 65 247–256. 10.1111/j.1541-0420.2008.01049.x 18479482 PMC2776072

[B4] Berber T. Alpkocak A. Balci P. Dicle O. (2013). Breast mass contour segmentation algorithm in digital mammograms. *Comput. Methods Progr. Biomed.* 110 150–159. 10.1016/j.cmpb.2012.11.003 23273502

[B5] Bornefalk H. Hermansson A. B. (2005). On the comparison of FROC curves in mammography CAD systems. *Med. Phys.* 32 412–417. 10.1118/1.1844433 15789587

[B6] Carney P. A. Miglioretti D. L. Yankaskas B. C. Kerlikowske K. Rosenberg R. Rutter C. M. (2003). Individual and combined effects of age, breast density, and hormone replacement therapy use on the accuracy of screening mammography. *Ann. Intern. Med.* 138 168–175. 10.7326/0003-4819-138-3-200302040-00008 12558355

[B7] Checka C. M. Chun J. E. Schnabel F. R. Lee J. Toth H. (2012). The relationship of mammographic density and age: implications for breast cancer screening. *Am. J. Roentgenol.* 198 W292–W295. 10.2214/AJR.10.6049 22358028

[B8] Chong A. Weinstein S. P. McDonald E. S. Conant E. F. (2019). Digital breast tomosynthesis: concepts and clinical practice. *Radiology* 292 1–14. 10.1148/radiol.2019180760 31084476 PMC6604796

[B9] Conant E. F. Barlow W. E. Herschorn S. D. Weaver D. L. Beaber E. F. Tosteson A. N. A. (2019). Association of digital breast tomosynthesis vs digital mammography with cancer detection and recall rates by age and breast density. *JAMA Oncol.* 5 635–642. 10.1001/jamaoncol.2018.7078 30816931 PMC6512257

[B10] Fan M. Li Y. Zheng S. Peng W. Tang W. Li L. (2019). Computer-aided detection of mass in digital breast tomosynthesis using a faster region-based convolutional neural network. *Methods* 166 103–111. 10.1016/j.ymeth.2019.02.010 30771490

[B11] Fontaine M. Tourasse C. Pages E. Laurent N. Laffargue G. Millet I. (2019). Local tumor staging of breast cancer: digital mammography versus digital mammography plus tomosynthesis. *Radiology* 291 594–603. 10.1148/radiol.2019182457 30964425

[B12] Geras K. J. Mann R. M. Moy L. (2019). Artificial intelligence for mammography and digital breast tomosynthesis: current concepts and future perspectives. *Radiology* 293 246–259. 10.1148/radiol.2019182627 31549948 PMC6822772

[B13] Haas B. M. Kalra V. Geisel J. Raghu M. Durand M. Philpotts L. E. (2013). Comparison of tomosynthesis plus digital mammography and digital mammography alone for breast cancer screening. *Radiology* 269 694–700. 10.1148/radiol.13130307 23901124

[B14] He K. Gkioxari G. Dollár P. Girshick R. (2020). Mask R-CNN. *IEEE Trans. Patt. Anal. Mach. Intellig.* 42 386–397. 10.1109/TPAMI.2018.2844175 29994331

[B15] He K. Zhang X. Ren S. Sun J. (2015). “Delving deep into rectifiers: surpassing human-level performance on imagenet classification,” in *Proceedings of the 2015 IEEE International Conference on Computer Vision (ICCV)*, Santiago, 1026–1034.

[B16] He K. Zhang X. Ren S. Sun J. (2016). “Deep residual learning for image recognition,” in *Proceedings of the IEEE Conference on Computer Vision and Pattern Recognition*, San Juan, 770–778.

[B17] Hofvind S. Hovda T. Holen A. S. Lee C. I. Albertsen J. Bjorndal H. (2018). Digital breast tomosynthesis and synthetic 2D mammography versus digital mammography: evaluation in a population-based screening program. *Radiology* 287 787–794. 10.1148/radiol.2018171361 29494322

[B18] Kingma D. Ba J. (2014). “Adam: a method for stochastic optimization,” in *Proceedings of the International Conference on Learning Representations*, Vancouver, BC.

[B19] Kus P. Karagoz I. (2012). Fully automated gradient based breast boundary detection for digitized X-ray mammograms. *Comput. Biol. Med.* 42 75–82. 10.1016/j.compbiomed.2011.10.011 22118773

[B20] Lai X. Yang W. Li R. (2020). DBT masses automatic segmentation using U-net neural networks. *Comput. Math. Methods Med.* 2020:7156165. 10.1155/2020/7156165 32411285 PMC7204342

[B21] Li X. Qin G. G. He Q. Sun L. Zeng H. He Z. L. (2019). Digital breast tomosynthesis versus digital mammography: integration of image modalities enhances deep learning-based breast mass classification. *Eur. Radiol.* 30 778–788. 10.1007/s00330-019-06457-5 31691121

[B22] Lin T. Dollár P. Girshick R. He K. Hariharan B. Belongie S. (2017). “Feature pyramid networks for object detection,” in *Proceedings of the 2017 IEEE Conference on Computer Vision and Pattern Recognition (CVPR)*, Honolulu, HI, 936–944.

[B23] Michell M. J. Iqbal A. Wasan R. K. Evans D. R. Peacock C. Lawinski C. P. (2012). A comparison of the accuracy of film-screen mammography, full-field digital mammography, and digital breast tomosynthesis. *Clin. Radiol.* 67 976–981. 10.1016/j.crad.2012.03.009 22625656

[B24] Nystrom L. Andersson I. Bjurstam N. Frisell J. Nordenskjold B. Rutqvist L. E. (2002). Long-term effects of mammography screening: updated overview of the Swedish randomised trials. *Lancet* 359 909–919. 10.1016/S0140-6736(02)08020-0 11918907

[B25] Osteras B. H. Martinsen A. C. T. Gullien R. Skaane P. (2019). Digital mammography versus breast tomosynthesis: impact of breast density on diagnostic performance in population-based screening. *Radiology* 293 60–68. 10.1148/radiol.2019190425 31407968

[B26] Pohlmann S. T. L. Lim Y. Y. Harkness E. Pritchard S. Taylor C. J. Astley S. M. (2017). Three-dimensional segmentation of breast masses from digital breast tomosynthesis images. *J. Med. Imaging* 4:034007 10.1117/1.JMI.4.3.034007 PMC5603772 28948195

[B27] Reiser I. Nishikawa R. M. Giger M. L. Wu T. Rafferty E. A. Moore R. (2006). Computerized mass detection for digital breast tomosynthesis directly from the projection images. *Med. Phys.* 33 482–491. 10.1118/1.2163390 16532956

[B28] Ren S. He K. Girshick R. Sun J. (2017). Faster R-CNN: towards real-time object detection with region proposal networks. *IEEE Trans. Pattern Anal. Mach. Intell.* 39 1137–1149. 10.1109/TPAMI.2016.2577031 27295650

[B29] Ribli D. Horvath A. Unger Z. Pollner P. Csabai I. (2018). Detecting and classifying lesions in mammograms with deep learning. *Sci. Rep.* 8:4165. 10.1038/s41598-018-22437-z 29545529 PMC5854668

[B30] Samala R. K. Chan H. P. Hadjiiski L. Helvie M. A. Wei J. Cha K. (2016). Mass detection in digital breast tomosynthesis: deep convolutional neural network with transfer learning from mammography. *Med. Phys.* 43:6654 10.1118/1.4967345 PMC5135717 27908154

[B31] Samala R. K. Chan H. P. Hadjiiski L. M. Helvie M. A. Richter C. Cha K. (2018). Evolutionary pruning of transfer learned deep convolutional neural network for breast cancer diagnosis in digital breast tomosynthesis. *Phys. Med. Biol.* 63:e095005. 10.1088/1361-6560/Aabb5b 29616660 PMC5967610

[B32] Samala R. K. Heang-Ping C. Hadjiiski L. Helvie M. A. Richter C. D. Cha K. H. (2019). Breast cancer diagnosis in digital breast tomosynthesis: effects of training sample size on multi-stage transfer learning using deep neural nets. *IEEE Trans. Med. Imaging* 38 686–696. 10.1109/TMI.2018.2870343 31622238 PMC6812655

[B33] Skaane P. Bandos A. L. Niklason L. T. Sebuodegard S. Osteras B. H. Gullien R. (2019). Digital mammography versus digital mammography plus tomosynthesis in breast cancer screening: the oslo tomosynthesis screening trial. *Radiology* 291 22–29. 10.1148/radiol.2019182394 30777808

[B34] Tagliafico A. S. Calabrese M. Bignotti B. Signori A. Fisci E. Rossi F. (2017). Accuracy and reading time for six strategies using digital breast tomosynthesis in women with mammographically negative dense breasts. *Eur. Radiol.* 27 5179–5184. 10.1007/s00330-017-4918-5 28643094

[B35] Varela C. Timp S. Karssemeijer N. (2006). Use of border information in the classification of mammographic masses. *Phys. Med. Biol.* 51 425–441. 10.1088/0031-9155/51/2/016 16394348

[B36] Vourtsis A. Berg W. A. (2019). Breast density implications and supplemental screening. *Eur. Radiol.* 29 1762–1777. 10.1007/s00330-018-5668-8 30255244 PMC6420861

[B37] Wu Y. T. Zhou C. Chan H. P. Paramagul C. Hadjiiski L. M. Daly C. P. (2010). Dynamic multiple thresholding breast boundary detection algorithm for mammograms. *Med. Phys.* 37 391–401. 10.1118/1.3273062 20175501 PMC2809702

[B38] Yousefi M. Krzyzak A. Suen C. Y. (2018). Mass detection in digital breast tomosynthesis data using convolutional neural networks and multiple instance learning. *Comput. Biol. Med.* 96 283–293. 10.1016/j.compbiomed.2018.04.004 29665537

[B39] Zhang C. Li L. (2014). *3D Segmentation of Masses in DCE-MRI Images Using FCM and Adaptive MRF.* San Diego, CA: SPIE.

[B40] Zhang Y. Chan H. P. Sahiner B. Wei J. Goodsitt M. M. Hadjiiski L. M. (2006). A comparative study of limited-angle cone-beam reconstruction methods for breast tomosynthesis. *Med. Phys.* 33 3781–3795. 10.1118/1.2237543 17089843 PMC2728559

