# Supplementary material for: Mass Detection and Segmentation in Digital Breast Tomosynthesis Using 3D-Mask Region-Based Convolutional Neural Network: A Comparative Analysis
Source: Front Mol Biosci. 2020 Nov 11;7:599333. doi: 10.3389/fmolb.2020.599333 (PMC7686533; doi:10.3389/fmolb.2020.599333)
Supplement: Supplementary file 1 [file Table_1.DOCX]

| Feature pyramid network (FPN) | 2D |  |
| --- | --- | --- |
|  | 3D |  |
| Region proposal network (RPN) | 2D |  |
|  | 3D |  |
| Detection batch | 2D |  |
|  | 3D |  |
| Mask  Branch | 2D |  |
|  | 3D |  |

Supplementary Figure 1. The 2D- and 3D-Mask RCNN network structures

Supplementary Table 1. Hyperparameters of the networks

| Name | Batch size | RPN train anchors | Epochs | Learning rate | Backbone |
| --- | --- | --- | --- | --- | --- |
| Faster RCNN | 2 | 256 | 200 | 0.001 | VGG-16 |
| Mask RCNN | 2 | 128 | 200 | 0.001 | ResNet-50 |
| 3D-Mask RCNN | 4 | 32 | 200 | 0.0001 | 3D ResNet-50 |

Note: RPN = region proposal network; RPN train anchors refers to the number of anchors used for RPN training per image/volume.

Supplementary Table 2. Comparison of the mass detection performances of the 3D-Mask RCNN, 2D-Mask RCNN and Faster RCNN

| 3D-Mask RCNN | Confidence intervals | *P* value |
| --- | --- | --- |
| 2D-Mask RCNN | (0.2511,0.3229) | 0.005 |
| Faster RCNN | (0.4444,0.4960) | 0.007 |
